# A Comparison Between Skeletal Class II and Class III Malocclusion Patients in Terms of the Masticatory Muscles’ Activity: A Cross-Sectional Study

**DOI:** 10.7759/cureus.59861

**Published:** 2024-05-08

**Authors:** Ali A. Saker, Mudar Mohammad Mousa, Mohammad Y Hajeer, Ibrahim Haddad, Jacqueline Bashar Alhaffar, Mohamed Youssef

**Affiliations:** 1 Department of Orthodontics, Faculty of Dentistry, University of Damascus, Damascus, SYR; 2 Department of Basic Sciences, Faculty of Dentistry, University of Damascus, Damascus, SYR; 3 Department of Oral and Maxillofacial Surgery, Faculty of Dentistry, University of Damascus, Damascus, SYR

**Keywords:** electromyography (emg), emg, muscular activity, class iii patients, class ii patients, orbicularis oris muscle, digastric muscle, buccinator muscle, masseter muscle, temporalis muscle

## Abstract

Background

This study aimed to determine if individuals with skeletal Class II and skeletal Class III malocclusions had different levels of masticatory muscle activity.

Materials and methods

This cross-sectional study, conducted at the University of Damascus, investigated the myoelectric activity of perioral muscles in patients with Class II and III malocclusions. The sample size of 60 patients was determined according to a prior sample size calculation. Patients were selected based on specific inclusion and exclusion criteria and divided into Class II and III groups. Electromyography was used to monitor the activity of various muscles, including the temporalis, masseter, orbicularis oris, buccinator, mentalis, and digastric muscles.

Results

The study found similar muscle activity within the same group in the temporalis, masseter, buccinator, digastric, and orbicularis oris muscles. No significant differences were observed between the Class II and III groups for several oral and perioral muscles (P > 0.05). However, the mean activity of the digastric muscle was significantly greater in the Class II group (P < 0.05), whereas the mean activity of the mentalis muscle was smaller in the Class II group (P < 0.05).

Conclusions

Perioral muscles influence facial complex development and jaw relationship, affecting orthodontic treatment. Digastric muscle activity is greater in Class II patients, while mental muscle activity is smaller in Class III patients. Further studies are needed for older age groups and other skeletal malocclusion types.

## Introduction

Class II and III malocclusions are disorders in the sagittal direction of the bony bases of both the upper and lower jaws. These disorders may arise from a problem located in either or both the upper and lower dental arches, as well as from a positional or dimensional disorder [1,2].

Successful treatment of malocclusion necessitates an understanding of the causative pathogenetic factors. Opinions about the true causes of malocclusion are multiple and contradictory. Knowing these causes is the first step toward a proper diagnosis [3], the decisive and fundamental step. One can treat or even prevent disease conditions by avoiding and modifying these causes [4]. Several contradictory theories and opinions have emerged to determine the causes of malocclusion and dentofacial disorders, some of which focus on the role of genetic factors as a decisive factor in the development of the dentofacial complex, and others focus on environmental factors in general and functional factors in particular [5].

Fränkel suggested that the mass of soft tissues and active muscles exerts a restraining effect on the dental arches’ outward development, counteracting the tongue’s pressure on the teeth [6]. Here, the teeth take their final position through the balance between the external and internal oral forces, and the abnormal function of the perioral muscles can prevent reaching the ideal model of growth and development [6].

Ueda et al. found a strong relationship between face morphology and myoelectric activity after examining the electrical activity of the gastrocnemius, masseter, and temporalis muscles in several individuals with various facial patterns [7].

Additionally, Vianna-Lara et al. discovered a correlation between the development pattern and the myoelectric activity of the masseter and temporalis [8]. However, Custodio et al. found a rise in myoelectric activity with a horizontal growth pattern [9]. Ramsundar et al. found that the degree of muscle activity during chewing and at rest was unaffected by the protrusion associated with Class II malocclusion [10]. Alabdullah et al. identified a significant relationship between the activity of facial muscles and the pattern of facial growth [11]. Saker et al. compared the electromyographic activity between Class I and Class II skeletal malocclusion in their study, and they found significant differences between Class I and Class II malocclusion in muscle activity [3].

The causes of malocclusion continue to be debated, focusing on genetic and environmental factors, particularly those related to function. The research question was, “Is there a difference in the muscular activity of the masticatory muscles between individuals with skeletal Class II and skeletal Class III malocclusion?” given the importance of masticatory system-related muscular activity in developing the dental-facial complex.

## Materials and methods

Study design and settings

This was a cross-sectional study of patients referred to the Department of Orthodontics at the University of Damascus. The electrical activity of the perioral muscles was documented at the Faculty of Dentistry’s Department of Biological Sciences at the University of Damascus. The protocol for this study was evaluated and approved by the Local Research Ethics Committee of the Faculty of Dentistry, University of Damascus (approval number UDDS-254-2024HG/SRC1843). The University of Damascus funded this study (ref: 501100020595).

Sample size estimation

The G-Power 3.1© software (Heinrich-Heine-University, Düsseldorf, Germany) was utilized to determine the required sample size for the study. An effect size of 0.34 µv was considered the smallest clinically significant difference between the two groups regarding myoelectric activity [12]. Using an independent samples t-test with a significance level of 0.05 and a power of 85%, the acceptable sample size was 60 patients.

Patient recruitment

A total of 183 Damascus University Department of Orthodontics archive patients underwent a clinical assessment. Moreover, 53 Class II and 42 Class III patients have fulfilled the inclusion criteria. Based on the sample size, 60 patients were chosen from the sampling frame and divided into two equal groups based on their skeletal relationships in the sagittal plane as follows:

The first group included 30 patients with normal skeletal Class II malocclusion, and the second group included 30 patients with skeletal Class III malocclusion. Each participant or their parent read a written information sheet, and a consent form was signed. Patients were selected for the study based on the following inclusion and exclusion criteria:

Inclusion criteria included patients within the age range of 11-14 years, with normal facial height as evaluated by a Bjork sum of 396 ± 6°, exhibiting a typical overbite of not less than 2 mm and not exceeding one-third of the length of the lower incisor crowns, and without posterior crossbite. Patients in the skeletal Class III group had a midsagittal plane ANB angle of ≤1°. Patients in the skeletal Class II group exhibited a Class II division 1 malocclusion, characterized by an overjet exceeding 5 mm. Additionally, they demonstrated a Class II skeletal relationship in the midsagittal plane, with an ANB angle surpassing 4º.

Exclusion criteria included crowding (Little’s Irregularity Index ≥3), developmental abnormalities, soreness of facial muscles, temporomandibular joint diseases, scars or burns on face tissues, and history of orthodontic treatment.

Electromyography (EMG)

Initially, the patient was positioned to sit on the dental chair, with their head upright and the Frankfurt plane parallel to the ground or horizon. Every patient was expected to adopt this position as a baseline. The patient’s head was rested on the back of the chair, and EMG was conducted based on this position. The temporalis, masseter, orbicularis, buccinator, mentalis, and digastric muscles were identified by touch [10]. The skin area was cleaned with alcohol, and the forehead was also cleaned with alcohol to establish a connection with the GND ground electrode, per the guidelines.

A specially prepared gel was applied to the funnel-shaped electrodes, which were then attached to the muscle under examination using a medical adhesive. This guarantees the stability of the electrode throughout the planning process, with the electrodes of one unit being 2 cm apart from each other. This procedure was performed symmetrically on both sides to obtain muscle recordings from both sides, except for the chin muscle, which was treated as a single muscle.

**Figure 1:**
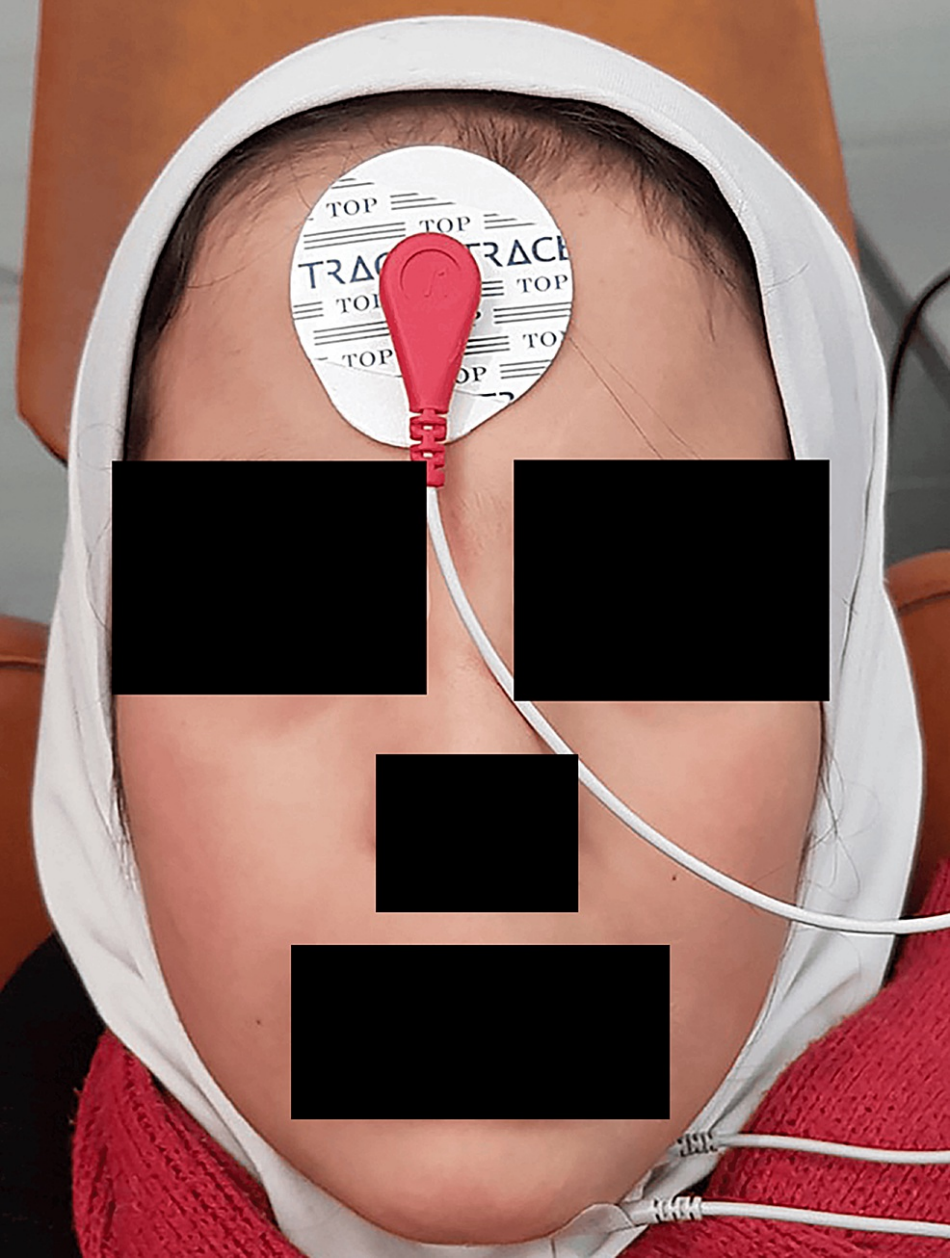
Connecting the ground electrode (GND) to the forehead before recording muscle activity

The ground electrode (GND) was attached to the forehead (Figure 1), and the program started. The planning type and location, specific muscle, and electrode type were chosen, and recording commenced in each position for five seconds - two seconds before issuing the command and two seconds following it, following the program’s settings.

**Figure 2:**
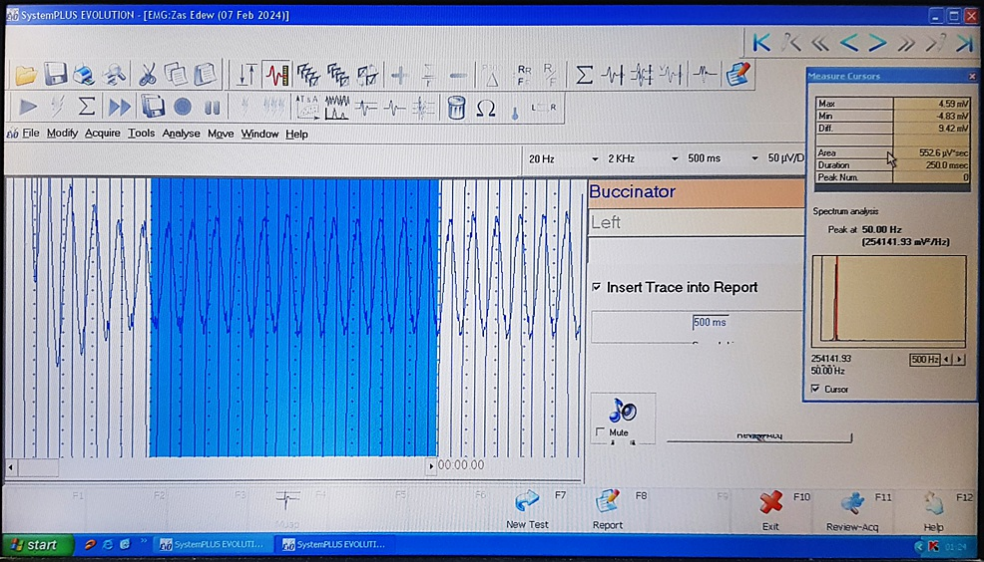
Recording the muscular activity using a specific electromyographic software

The recording command was initiated by pressing the space bar on the keyboard. To prevent recording errors, planning was executed in a particular order and under certain program conditions. In the program settings, the first track was assigned to the right side and the second to the left. Then, the “spontaneous” option was selected for the resting position (as shown in Figure 2).

Statistical analysis

Fundamental statistical analyses were performed using IBM SPSS Statistics for Windows, Version 23.0 (Released 2015; IBM Corp., Armonk, NY, USA). At the outset, the distribution of all variables used in the study, whether normal or not, was scrutinized using the Kolmogorov-Smirnov test to determine normality. The independent sample t-test was used to compare the two groups to see if the data conformed to a normal distribution. In cases where the data did not follow a normal distribution, the Mann-Whitney U test was employed to detect any significant differences between the two groups. The Wilcoxon test was also implemented to investigate any notable differences between the right and left muscles.

## Results

The study findings indicated that muscle activity (specifically in the temporalis, masseter, buccinator, digastric, and orbicularis oris muscles) exhibited similarity within the same group between the right and left sides. Notably, there were no statistically significant differences observed for any of these muscles (P > 0.05), as shown in Table 1 and Table 2.

**Table 1:** Descriptive statistics of muscle activity on both sides in the Class II malocclusion group and the significance of the differences between the two sides ^a^ Paired samples t-test ^b^ Wilcoxon signed-ranks test

		Right (n = 30)		Left (n = 30)		Mean difference	95% confidence interval		P-value
		Mean ± SD	Median	Mean ± SD	Median		Lower	Upper	
Temporalis muscle	MIN	-39.32 ± 17.5	-32.4	-41.02 ± 24.86	-37.35	1.7	-8.48	11.89	0.813^b^
	MAX	41.01 ± 18.33	32.25	36.96 ± 15.63	32.9	4.06	-2.24	10.36	0.681^b^
	OVERALL	80.33 ± 35.56	63.8	77.98 ± 37.06	69.4	2.35	-13.06	17.77	0.984^b^
Masseter muscle	MIN	-36.59 ± 10.81	-34.4	-37.58 ± 17.16	-33.15	0.99	-6.48	8.45	0.959^b^
	MAX	42.7 ± 19.29	34.3	40.38 ± 19.96	32.8	2.33	-6.42	11.07	0.629^b^
	OVERALL	79.29 ± 26.46	70.35	77.95 ± 35.35	66.9	1.34	-13.23	15.91	0.544^b^
Buccinator muscle	MIN	-48.05 ± 16.4	-43.75	-45.09 ± 13.2	-42.4	-2.96	-8.69	2.77	0.910^b^
	MAX	48.91 ± 14.21	47.2	45.04 ± 12.19	42.8	3.87	-1.18	8.92	0.711^b^
	OVERALL	96.96 ± 29.91	89.85	90.13 ± 22.37	86.95	6.83	-3.05	16.71	0.918^b^
Digastric muscle	MIN	-51.89 ± 16.64	-49.15	-50.18 ± 12.91	-49.5	-1.71	-7.6	4.18	0.558^a^
	MAX	51.47 ± 18.74	48.6	49.65 ± 12.83	49.25	1.82	-4.65	8.29	0.570^a^
	OVERALL	103.36 ± 31.59	99.35	99.83 ± 24.96	98.65	3.53	-6.98	14.03	0.399^b^
Orbicularis oris muscle	MIN	-31.5 ± 6.82	-31.05	-28.32 ± 9.04	-26.4	-3.18	-7.2	0.85	0.117^a^
	MAX	32.56 ± 7.82	32.4	32.51 ± 10.83	29.45	0.05	-4.63	4.73	0.897^b^
	OVERALL	64.06 ± 11.93	63.9	60.83 ± 14.53	57.95	3.22	-2.32	8.77	0.289^b^

**Table 2:** Descriptive statistics of muscle activity on both sides in the Class III malocclusion group and the significance of the differences between the two sides ^a^ Paired samples t-test ^b^ Wilcoxon signed-ranks test

		Right (n = 30)		Left (n = 30)		Mean difference	95% confidence interval		P-value
		Mean ± SD	Median	Mean ± SD	Median		Lower	Upper	
Temporalis muscle	MIN	-44.33 ± 24.61	-37.5	-43.04 ± 19.19	-39.15	-1.29	-5.96	3.39	0.967^b^
	MAX	43.99 ± 23.02	37.6	43.67 ± 17.48	40.85	0.33	-4.71	5.36	0.805^b^
	OVERALL	88.32 ± 46.62	76.6	86.71 ± 35.63	83.95	1.61	-7.19	10.42	0.943^b^
Masseter muscle	MIN	-32.73 ± 24.62	-32.95	-32.53 ± 15.56	-31.65	-0.2	-10.94	10.54	0.837^b^
	MAX	44.01 ± 21.55	35.45	47.51 ± 35.28	35	-3.5	-15.13	8.13	0.559^b^
	OVERALL	76.74 ± 32.54	69.85	80.04 ± 36.22	70	-3.3	-14.13	7.53	0.484^b^
Buccinator muscle	MIN	-42.69 ± 12.22	-40.5	-42.93 ± 12.81	-41.4	0.24	-5.91	6.38	0.565^b^
	MAX	43.55 ± 12.61	40.25	43.81 ± 12.53	43.95	-0.26	-5.81	5.29	0.922^b^
	OVERALL	86.24 ± 24.39	81.65	86.74 ± 23.67	86.05	-0.49	-11.42	10.44	0.861^b^
Digastric muscle	MIN	-36.63 ± 10.49	-36.1	-33.36 ± 6.75	-33.15	-3.26	-7.7	1.17	0.280^b^
	MAX	34.97 ± 10.43	35.05	34.56 ± 7.57	35.2	0.41	-3.86	4.69	0.510^b^
	OVERALL	71.6 ± 20.29	71.4	67.92 ± 12.66	70.6	3.68	-4.45	11.8	0.362^a^
Orbicularis oris muscle	MIN	-29.42 ± 10.39	-30.25	-30.29 ± 9.38	-30	0.87	-1.53	3.27	0.466^a^
	MAX	30.05 ± 9.62	29.1	30.95 ± 9.76	33.2	-0.9	-2.97	1.16	0.378^a^
	OVERALL	59.47 ± 17.71	61.9	61.24 ± 15.11	62.85	-1.77	-4.79	1.25	0.240^a^

There were no statistically significant differences in the activity of temporalis, masseter, buccinator, and orbicularis oris muscles between the Class II and Class III groups (P < 0.05; Table 3). However, the mean activity of the digastric muscle was significantly greater in the Class II group (101.59 µv ± 24.75 µv) than in the Class III group (69.76 µv ± 12.95 µv, P < 0.001). Similarly, the mean activity of the mentalis muscle was also significantly smaller in the Class II group (70.96 µv ± 18.6 µv) compared with the Class III group (78.74 µv ± 17.34 µv, P = 0.034).

**Table 3:** Descriptive statistics of muscle effectiveness in the two malocclusion groups and the significance of the difference between the two groups ^a^ Independent samples t-test ^b^ Mann-Whitney U test ^*^ Significant at the 0.05 level

		Class II group (n = 30)		Class III group (n = 30)		Mean difference	95% confidence interval		P-value
		Mean ± SD	Median	Mean ± SD	Median		Lower	Upper	
Temporalis muscle	MIN	-40.17 ± 16.61	-37.55	-43.69 ± 21.16	-37.95	3.52	-6.31	13.35	0.790^b^
	MAX	38.99 ± 14.8	32.45	43.83 ± 19.29	40.58	-4.85	-13.73	4.04	0.271^b^
	OVERALL	79.15 ± 29.88	73.2	87.52 ± 39.78	78.48	-8.36	-26.55	9.82	0.515^b^
Masseter muscle	MIN	-37.08 ± 10.29	-34.4	-32.63 ± 14.75	-33.30	-4.45	-11.03	2.12	0.399^b^
	MAX	41.54 ± 15.75	36.45	45.76 ± 24.74	37.75	-4.22	-14.94	6.5	0.530^b^
	OVERALL	78.62 ± 24.38	70.7	78.39 ± 31.22	70.05	0.23	-14.25	14.71	0.712^b^
Buccinator muscle	MIN	-46.57 ± 12.76	-45.075	-42.81 ± 9.43	-42.78	-3.77	-9.56	2.03	0.337^b^
	MAX	46.97 ± 11.38	44.825	43.68 ± 10.14	42.98	3.29	-2.28	8.86	0.271^b^
	OVERALL	93.55 ± 22.86	90.775	86.49 ± 19.06	86.38	7.06	-3.82	17.93	0.237^b^
Digastric muscle	MIN	-51.04 ± 12.63	-49.1	-35 ± 6.53	-34.85	-16.04	-21.28	-10.81	<0.001^a^*
	MAX	50.56 ± 13.52	48.8	34.76 ± 7.09	35.83	15.79	10.18	21.41	<0.001^a^*
	OVERALL	101.59 ± 24.75	99.05	69.76 ± 12.95	72.33	31.84	21.56	42.11	<0.001^a^*
Orbicularis oris muscle	MIN	-29.91 ± 5.92	-29.85	-29.86 ± 9.36	-31.03	-0.05	-4.11	4.01	<0.001^a^*
	MAX	32.54 ± 7.06	32.6	30.5 ± 9.29	31.38	2.04	-2.23	6.3	0.343^a^
	OVERALL	62.45 ± 11.02	59.95	60.36 ± 15.96	62.13	2.09	-5	9.17	0.558^a^
Mentalis muscle	MIN	-34.24 ± 9.35	-33.6	-37.86 ± 9.84	-38.25	3.62	-1.34	8.58	0.150^a^
	MAX	36.71 ± 10.34	34.4	40.88 ± 10.65	38.15	-4.16	-9.59	1.26	0.053^b^
	OVERALL	70.96 ± 18.6	67.2	78.74 ± 17.34	75.7	-7.78	-17.07	1.51	0.034^b^*

## Discussion

The resting position of the mandible is considered one of the most important static positions to analyze because it can be evaluated repeatedly using an EMG device. The resting position of the lower jaw results from the dynamic balance between the synergistic and antagonistic muscles of the orofacial group in its primary tonic; this situation results from the muscles opposing the force of gravity only [13].

The clinical rest position is an active muscular state due to the harmony of the involved muscles. In the case of proper occlusion, this activity should be as low as possible [14]. The application of EMG signals in the research of facial muscles has garnered substantial attention. The electrical activity within the orofacial and periorificial muscles not only influences bone growth but also significantly impacts the duration and stability of orthodontic treatments [15].

There were no significant differences between the right and left sides regarding the electrical activity of all the studied muscles. This study’s results agreed with Saker et al.’s study [3], while the results of the current study differed from the results of Rahmawati et al.’s study [16]. This could be attributed to the absence of a one-sided chewing pattern in most participants in this study. Bakke demonstrated in his study conducted in 1993 that the presence of a unilateral chewing habit could lead to an increase in the size of the muscles on the side of chewing and, thus, an increase in the electrical activity of the muscles on this side [17].

No significant differences were observed between the two groups in the electrical activity of the masseter and temporalis muscles in the resting position. This can be explained by the fact that the effect of those muscles on the position of the lower jaw in the sagittal direction is limited, as its greatest effect is in the vertical direction [18]. The mean muscle activity of the digastric muscle was significantly greater in Class II compared to Class I and Class III (P > 0.05). This indicates a role for this muscle in the posterior positioning of the mandible, and the results of this study agree with the study of Petrović et al. [19].

The mean muscle activity of the mental muscle was significantly greater in Class III malocclusion compared to Class II malocclusion (P < 0.05). The mental muscle is the primary muscle for closing the lips [20]. The results of the current study agreed with the findings of Dutra et al. [21].

Strengths and limitations

Muscle activity was assessed at only one point, using the resting position. Future research should explore the electrical activity of the muscles in various positions following the correction of skeletal malocclusion. Another limitation of the current study is the relatively young age of the sample. This is primarily due to Class III patients seeking treatment at earlier ages than other types of malocclusion.

## Conclusions

The perioral muscles play a crucial role in shaping the facial structures and determining the jaw relationship, which should be considered when planning orthodontic treatment. The electrical activity of the digastric muscle was greater in skeletal Class II patients compared to skeletal Class III ones. The electrical activity of the mental muscle in Class II patients was smaller than that of Class III patients. There is a need for further studies on the electrical activity of the examined muscles in older age groups and different types of skeletal malocclusion.
